# Molecular Detection and Characterization of *Babesia* and *Theileria* in Cattle and Water Buffaloes from Southern Luzon, Philippines

**DOI:** 10.3390/microorganisms10040678

**Published:** 2022-03-22

**Authors:** Ian Cary B. Prado, Larry Xerxes B. Capuno, Princess DLP. Collera, Aaron Paul D. Cabralda, Kristina Andrea S. De Ramos, John Michael G. Bernardo, Billy P. Divina, Tatsunori Masatani, Tetsuya Tanaka, Remil L. Galay

**Affiliations:** 1 Department of Veterinary Clinical Sciences, College of Veterinary Medicine, University of the Philippines Los Baños, Laguna 4031, Philippines; ibprado@up.edu.ph; 2 Department of Veterinary Paraclinical Sciences, College of Veterinary Medicine, University of the Philippines Los Baños, Laguna 4031, Philippines; capunol1606@uerm.edu.ph (L.X.B.C.J.); pdcollera1@up.edu.ph (P.D.C.); adcabralda@up.edu.ph (A.P.D.C.); kcsandalo@up.edu.ph (K.A.S.D.R.); jgbernardo@up.edu.ph (J.M.G.B.); bpdivina@up.edu.ph (B.P.D.); 3 Laboratory of Zoonotic Diseases, Faculty of Applied Biological Sciences, Gifu University, Yanagido 1-1, Gifu 501-1193, Japan; mstn@gifu-u.ac.jp; 4 Laboratory of Infectious Diseases, Joint Faculty of Veterinary Medicine, Kagoshima University, Korimoto 1-21-24, Kagoshima 890-0065, Japan

**Keywords:** *Babesia*, *Theileria*, piroplasms, tick-borne disease, cattle, water buffalo

## Abstract

*Babesia* and *Theileria* are tick-borne protozoan parasites that can cause significant economic losses in the cattle industry. This study aimed to contribute to the limited epidemiological data on *Theileria orientalis* as well as *Babesia bigemina* and *B. bovis* in large ruminants in the Philippines. Blood samples of 412 cattle and 108 water buffalo collected from four provinces in Southern Luzon, Philippines, were initially tested for the *18S rRNA* gene of piroplasms through nested PCR. Positive samples were further subjected to species-specific PCR. The *18s rRNA* of piroplasms was detected in 123 (29.9%) cattle and three (2.8%) water buffaloes. *Theileria orientalis* was found to be the most common piroplasm in cattle with a detection rate of 17.5%, followed by *Babesia bovis* and *B. bigemina*. Co-infections were also observed. Two water buffaloes were found infected with *B. bovis*, while one was positive for *B. bigemina*. The phylogenetic tree for *B. bovis* showed clustering of the isolates in two clades together with isolates from other countries, and a third separate clade. Meanwhile, the *T. orientalis* isolates in this study were distributed in three clades together with reported isolates from other countries. This study confirms the presence of *T. orientalis* in the Philippines and reports the genetic diversity of *B. bovis* and *T. orientalis*.

## 1. Introduction

Cattle and water buffaloes have an integral role in the livelihood of farmers in the Philippines. They are mostly raised in backyard farms to augment household income from animal sales [1,2,3] and/or integrated into crop-based farming systems where they are utilized for their draft power [3,4,5]. In terms of dairy production, crossbred cattle are used mainly in commercial systems [3]. With the establishment of the Philippine Carabao Center under the Department of Agriculture in 1996, exotic breeds of water buffaloes, such as the Murrah buffalo, have been imported for the production of fresh milk and milk-based local products, and to upgrade the Philippine carabao (local breed of water buffalo) for these purposes [5,6,7]. Programs for the importation of foreign stocks to upgrade indigenous breeds of cattle and water buffaloes are encouraged by government agencies in the country [3]. However, tick-borne diseases (TBDs) can negatively affect these development programs, and yet few studies are being conducted to monitor their local status [8]. 

Babesiosis and theileriosis, collectively known as piroplasmosis, are among the most important TBDs that incur huge economic losses in many regions of the world [9]. These diseases are listed by the World Organization for Animal Health as important diseases of livestock [10]. The causative agents, *Babesia* and *Theileria*, are protozoan parasites named after their characteristic pear-shaped or piriform intraerythrocytic stages [11]. Piroplasms infect both invertebrate and vertebrate hosts, with sexual reproduction occurring in the former, and asexual reproduction in the latter [11,12]. Hard ticks are the only identified vectors to date [11], but these parasites can infect a wide variety of mammals making their distribution quite extensive [11,12]. 

Babesiosis is an economically important blood-borne disease of free-living animals and is considered an emerging zoonosis in humans [12,13]. The cattle industry, in particular, is significantly affected by babesiosis (also known as cattle fever or tick fever) [13]. *Babesia bovis* and *B. bigemina* are primarily responsible for the disease in cattle in tropical and subtropical regions, including Asia, Africa, Australia, and the Americas [13,14,15]. Babesiosis is characterized by fever, icterus, and hemoglobinuria and, less commonly, acute encephalitis leading to deficits in movement, coma, and even sudden death [15]. Losses are incurred from the lowered productivity, mortality, and cost of treatment of affected cattle [13]. Water buffaloes with babesiosis may present similar signs as in cattle but may be less severe or milder in form [16,17]. 

Theileriosis is one of the major TBDs of domestic and wild bovids globally [18,19]. In the Asia-Pacific region, oriental theileriosis caused by the *T. orientalis* complex has been reported in cattle and water buffaloes [19]. The disease in cattle is often asymptomatic but may be severe when naive cattle are introduced to endemic areas [20,21]. These animals may develop signs related to hemolytic anemia of varying degrees of severity, as well as lethargy and anorexia [21]. Fatal disease outbreaks, in which hemolytic anemia is the predominant finding, may also be observed in water buffaloes after being subjected to stress during transportation [22]. Although clinical findings are useful in such acute cases, subclinical and long-standing carriers need to be tested by more accurate diagnostic tests [23]. 

Epidemiological studies have been conducted with the use of molecular techniques such as PCR [8,24]. The occurrence of *Theileria* in the Philippines, with a suggestion that it could be a new species, was only recently reported in cattle [25]. Hence, epidemiological data and information on the genetic diversity of *Theileria* in the country are still limited [8]. With the continuous growth of the local livestock industry, attention must be given to these pathogens, as they can cause diseases that can impede this improvement [26]. In this study, the presence of *B. bigemina, B. bovis,* and *T. orientalis* in cattle and water buffaloes from selected provinces in Southern Luzon, Philippines, was investigated through PCR. The data from this study provide information on the distribution of these blood parasites in the large ruminant population that will be helpful in the design and implementation of control and prevention strategies in the country.

## 2. Materials and Methods

### 2.1. Study Area and Blood Samples

**Figure 1:**
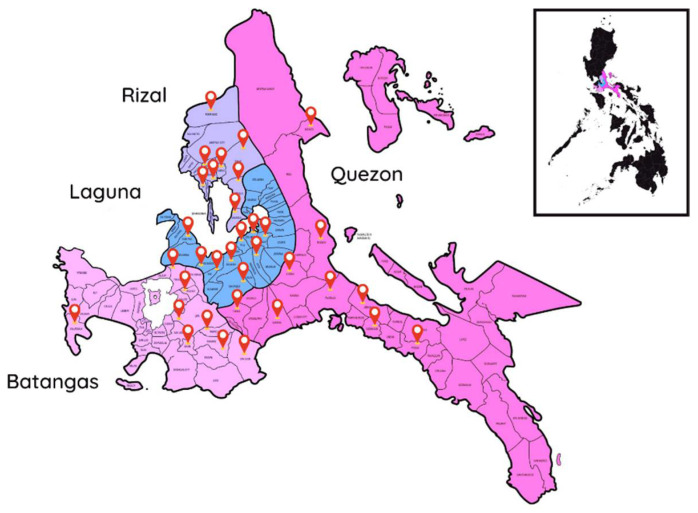
Map showing the four provinces of Region 4A (CALABARZON) included in the study, namely Laguna, Batangas, Rizal, and Quezon. Red pins mark the municipalities where the blood samples were collected. The labels and smaller map were placed using online graphic design platform Canva (https://www.canva.com/; accessed on 16 February 2022).

Blood samples previously collected from 412 cattle and 108 water buffaloes in the provinces of Laguna, Batangas, Rizal, and Quezon in Region 4A (CALABARZON), Philippines (Figure 1) between March 2016 and October 2019 were used in this study [27,28]. The annual mean temperature and relative humidity during the study period were 23.2 °C and 89%, respectively [29]. The region is known to have high populations of cattle and water buffaloes [1,2]. The animals were of various ages, sexes, and breed types. The majority were apparently healthy at the time of blood collection, as was described previously [27,28]. Ticks were not observed in farms applying acaricides, such as amitraz and ivermectin. In some farms, however, ticks were observed mostly in cattle with varying degrees of infestation, from mild (1−5 ticks) to very high tick infestations (over 50 ticks) [30]. All ticks were morphologically identified as *Rhipicephalus microplus* [27]. Total DNA was extracted from the blood samples using a commercial DNA extraction kit (innuPREP DNA/RNA Mini Kit, Analytik Jena, Jena, Germany) following the manufacturer’s instructions. 

### 2.2. PCR for Detection of Piroplasms

A nested PCR targeting the 18S ribosomal RNA (*18S rRNA*) gene was initially performed, as previously described [31], to detect the occurrence of piroplasms regardless of their genera. For specific detection of *Babesia* spp., amplification of a 211-bp fragment of the apical membrane antigen-1 (*AMA-1*) gene of *B. bigemina* and a 503-bp fragment of the spherical body protein-4 (*SBP-4)* gene of *B. bovis* were conducted by conventional and nested PCR, respectively, as previously described [32,33]. To detect *T. orientalis,* a conventional PCR for the 776-bp fragment of the major piroplasm surface protein (*MPSP*) gene was also conducted following a previous procedure [34].

The PCR mixtures consisted of 2× PCR buffer, 10 pmol each of forward and reverse primers, polymerase (Tks Gflex DNA Polymerase, TaKaRa, Shiga, Japan), nuclease-free water, and a template (DNA or first PCR product). The primers used in this study are shown in Table S1 in the supplementary materials, whereas the PCR conditions are listed in Table S2. DNA samples extracted from in vitro cultures of *B. bigemina* and *B. bovis,* and blood previously confirmed positive for *T. orientalis* were used as positive controls. Negative controls consisted of sterile ultrapure nuclease-free water. PCR products were electrophoresed in 2% agarose gel using 1× TAE buffer, stained with ethidium bromide, and visualized through a gel documentation system (Bio-Print, Vilber, Lourmat, France).

### 2.3. Sequencing of DNA and Analysis of Data

Twelve amplicons each of *B. bovis SBP-4* and *T. orientalis MPSP* were selected. These represented municipalities within the four provinces with positive samples. The selected amplicons were purified using a commercial kit (NucleoSpin Gel and PCR Clean-up kit, Macherey-Nagel, Duren, Germany) following the manufacturer’s instructions. After purification, the amplicons were submitted for capillary sequencing to a third-party laboratory using the corresponding forward primers for the conventional and nested PCR. The sequences of the selected isolates were then compared for similarity through multiple nucleotide sequence alignment in an online program (Clustal Omega, https://www.ebi.ac.uk/Tools/msa/clustalo/; accessed on 2 February 2022) [35]. Homologs of the sequences in previous isolates from other countries were obtained using BLAST (https://blast.ncbi.nlm.nih.gov/Blast.cgi; accessed on 2 February 2022) [36]. These were used to construct neighbor-joining phylogenetic trees following the Kimura 2-parameter method with 1000 bootstrap values of the MEGA software version 7. The sequences of selected isolates were deposited in the DNA Data Bank of Japan (accession numbers LC684833 to LC684846). 

The percent positives (number of positive samples divided by the number of examined samples per animal species × 100) were computed per province. Percentages of single and multiple infections of piroplasms in cattle were also calculated. Lastly, the presence of association of the occurrence of piroplasms and host attributes (species, type, and sex) was determined by chi-square analysis at a 95% confidence interval (α = 0.05) using the online software WinEpi^®^.

## 3. Results

Nested PCR for the detection of the *18S rRNA* of piroplasms (*Babesia/Theileria* spp.) in blood samples showed a total of 29.9% (123/412) positives in cattle and 2.8% (3/108) in water buffaloes (Table 1). Piroplasm-positive cattle blood samples came from the four provinces, with Batangas having the highest percent of positive cattle for piroplasms. No water buffalo samples were collected and tested from Rizal. Only 3 out of 108 blood samples (2.8%) from water buffaloes tested positive for piroplasms, and all came from the province of Quezon. These samples came from one male and two female water buffaloes of varying breed types (dairy, draft, and meat) and were more than a year old. 

Table 2 shows the number and percent of cattle blood samples positive for *B. bigemina, B. bovis,* and *T. orientalis.*

Monospecies infections, which were dominated by *T. orientalis* (14.1%), were the most common (22.6%) compared to co-infections with two (3.4%) or three (0.5%) species of piroplasms. Between the two species of *Babesia*, *B. bovis* was detected in more samples than *B. bigemina* (5.6% vs. 2.9%). All three piroplasms were detected in cattle from the provinces of Laguna, Batangas, and Quezon. In Rizal, where only *T. orientalis* was detected, the percent positive samples for this species were highest in this province. Different combinations of co-infections with two species occurred more in Batangas than in the other provinces. 

Interestingly, 14 out of 123 (11.4%) blood samples from cattle that were positive for piroplasms by nested PCR were negative for any species-specific PCR (data not shown). Only monospecies infections were detected in water buffalo blood samples. Two samples (1.9%) were *B. bovis*-positive, whereas one sample (0.9%) had *B. bigemina*.

Chi-square analysis showed that host species and type are significantly associated with the occurrence of at least one piroplasm (Table 3). There were higher percentages of cattle positive for piroplasms compared to water buffaloes, and likewise in dairy type compared to meat and draft types. Female animals appeared to have higher percent positives than males, but this difference was not significant.

**Figure 2:**
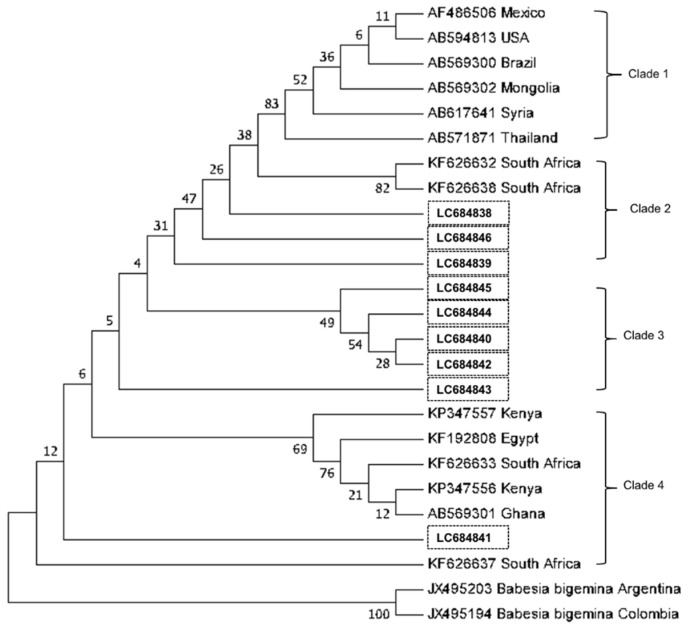
Neighbor-joining phylogenetic tree for *Babesia bovis* spherical body protein-4 constructed based on Kimura’s two-parameter substitution model. The numbers next to the branches indicate the percentage of replicate trees based on 1000 bootstrap replicates. The isolates from this study are enclosed in boxes.

Multiple sequence alignment of the *B. bovis SBP4* fragments showed that the twelve isolates had a similarity between 91–100% (data not shown). Four isolates from Batangas Province shared 99–100% identity; thus, a single representative was selected. This sequence and those of the remaining eight isolates were subjected to BLAST analysis, which revealed that 95–99% shared identity with the isolates from other countries. The phylogenetic tree in Figure 2 shows that the isolates in this study (accession numbers LC684838 to LC684846) belong to three clades. Three isolates clustered with two previously reported isolates from South Africa, and one isolate clustered with isolates from Kenya, Egypt, South Africa, and Ghana. Interestingly, five isolates from this study formed a separate clade, designated as clade 3.

**Figure 3:**
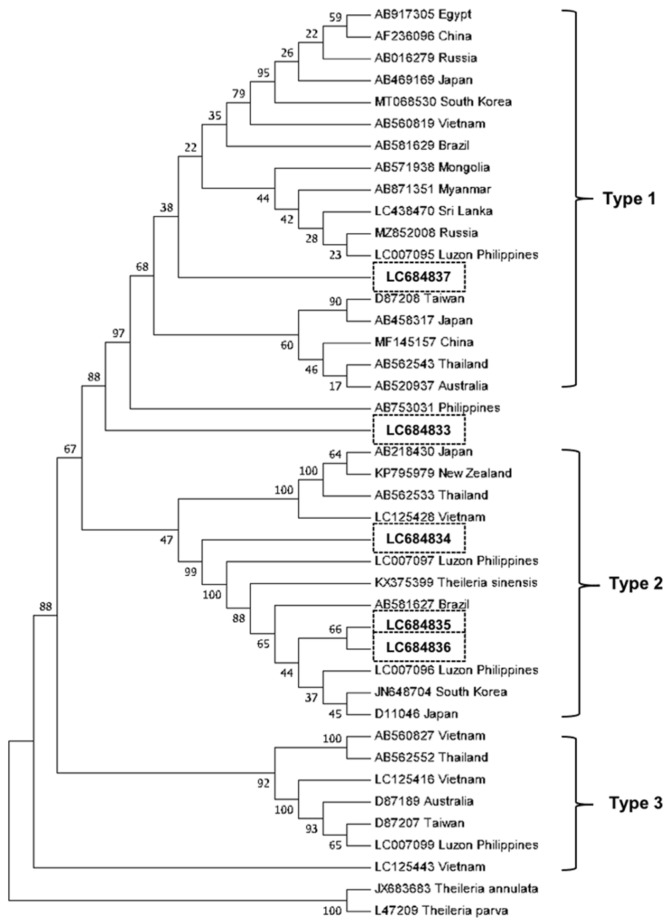
Phylogenetic tree for *Theileria orientalis* based on major piroplasm surface protein. Neighbor-joining method based on Kimura’s two-parameter substitution model was employed. The numbers next to the branches indicate the percentage of replicate trees based on 1000 bootstrap replicates. The isolates from this study are enclosed in boxes.

With regard to the sequenced amplicons of *T. orientalis MPSP* fragments, they shared 84–99% identity. BLAST analysis revealed a 91–99% homology with a previously reported *Theileria* spp. isolate from the Philippines and *T. orientalis, Theileria sergenti,* and *Theileria buffeli* isolates from other countries. Five isolates from this study (accession numbers LC684833 to LC684837) were included in the construction of a phylogenetic tree and were distributed to three clades (Figure 3). One isolate clustered with Type 1 isolates, including a previously reported isolate from Luzon, Philippines (GenBank accession number LC007095). Three isolates clustered with Type 2 isolates together with two previously reported isolates from Luzon, Philippines (GenBank accession numbers LC007096 and LC007097). Another isolate clustered with the first reported isolate of *Theileria* sp. from the Philippines (GenBank accession number AB753031) [25].

## 4. Discussion

Piroplasmosis, specifically babesiosis and theileriosis, are among the most widely distributed tick-borne protozoan diseases that cause devastation in the livestock industry [8,9]. The occurrence of *Babesia* in cattle has been extensively studied in several areas in the Philippines [8,27,36,37,38,39,40] but there is limited epidemiological data on *Theileria*. This study provides additional epidemiological data on *B. bigemina* and *B. bovis* in cattle and water buffaloes in Southern Luzon and confirms the presence of *T. orientalis* in cattle in the region through PCR.

Among the four provinces and three species of piroplasms, only *B. bigemina* was not detected in Rizal. This species was not detected in the Province of Batangas in a previous study [26] involving cattle in five provinces in the Philippines. In the said paper, a lower detection rate for *Babesia* was also reported, which could be due to the lower number of animals tested. Meanwhile, another study did not detect both *Babesia* and *Theileria* in Quezon [38], which may be due to the difference in the municipalities wherein samples were collected. Positive samples from cattle in this study were from the municipalities of Agdangan, Lucban, and Tiaong, whereas the positive samples for water buffaloes were obtained from the municipalities of Lucban, Pagbilao, and Pitogo. These areas were not sampled in the said study of Galon et al. [38]. In contrast to the results of this study, other studies in the Philippines reported a higher detection rate for *B. bigemina* than *B. bovis*. These include a study conducted in Cebu Island, which reported a detection rate of 15.4% and 10% for *B. bigemina* and *B. bovis*, respectively [40], and a study in Luzon that covered two dairy farms and obtained 61.6% and 45.4% detection rates for *B. bigemina* and *B. bovis*, respectively [39]. 

Previous studies in Southeast Asia also utilized PCR for the detection of *Babesia* in cattle [41,42,43,44,45]. Similar to our findings, a study in Indonesia, which also targeted the *B. bovis SBP-4* gene, reported a higher percent of positives for *B. bovis* (50.7%) than *B. bigemina* (19.1%) [41]. The same trend was reported in a study in Myanmar [42]. Conversely, a study in Thailand found that *B. bigemina* (13.1%) was more prevalent than *B. bovis* (5.5%) and attributed the difference to a higher chance of tick transmission of *B. bigemina* than *B. bovis* [43]. Two studies in Vietnam that also utilized PCR targeting the gene AMA-1 for *B. bigemina* found varying results. Weerasooriya et al. [44] found a higher detection rate for *B. bigemina* (10.9%) than *B. bovis* (8.9%) in cattle, whereas Sivakumar et al. [45] found a higher detection rate for *B. bovis* (8.9%) than *B. bigemina* (3.5%). These findings highlight the presence of geographic differences in the occurrence of piroplasms within a country and between countries and the need for forming measures to prevent TBDs for the protection of the cattle industry and trade in the region. 

A higher detection rate of piroplasms was observed in cattle (29.9%) than in water buffaloes (2.8%). As stated previously, all infected buffaloes were from the Province of Quezon, which also had the highest number of water buffaloes sampled among the four provinces. The results of species-specific PCR confirmed the presence of *B. bovis* in water buffaloes in Luzon, Philippines. However, this was lower than the reported 21% detection rate for *B. bovis* in water buffaloes in Bohol Island, Philippines [46]. The detection rate for *B. bigemina* in water buffaloes in the current study (0.9%) was also lower than the detection rate in an earlier report (4.4%), which covered mainly Luzon, Philippines [47]. Meanwhile, a study in a province in Central Luzon was not able to detect these blood parasites in water buffaloes [37]. This may be due to a smaller sample size compared to the other studies. In contrast to our findings, a study in Thailand reported a higher detection rate for *B. bovis* (11.2%) than *B. bigemina* (3.6%) in water buffaloes [33]. This is similar to a study in Vietnam, which reported a detection rate of 32.7% and 4.1% for *B. bovis* and *B. bigemina*, respectively. Moreover, the said study reported the detection of *T. orientalis* in water buffaloes in Vietnam [44], which is in contrast to negative results for the said piroplasm in this study. However, another study in Vietnam only detected *B. bovis* in water buffaloes (9.3%) [45]. 

The low detection rate of piroplasms in water buffaloes actually corroborates with our previous findings on the detection of other tick-borne pathogens [27,28], which is due to the lower susceptibility of water buffaloes to tick infestation. Their behavior of wallowing in muddy waters to regulate their body temperature, as well as their thick hide, contributed to the decreased tick attachment, consequently preventing the acquisition of these pathogens [28,48,49]. Nevertheless, the presence of *Babesia* still suggests the potential role of water buffaloes in the transmission of bovine babesiosis in the country [46]. In the Philippines and other Asian countries, water buffaloes may be pastured together or in the same area with cattle, particularly in backyard farm settings. Thus, our findings suggest that while no clinical manifestations might be observed, water buffaloes should also be monitored for TBDs that affect cattle [12,13]. In the current study, *B. bovis* and *B. bigemina* had a detection rate of 1.9% and 0.9% in water buffaloes, respectively. 

A significant finding in this study was the detection of *T. orientalis* in cattle from all four provinces. Two previous studies already reported the detection of *Theileria* in Luzon, including the Province of Laguna, but both utilized genus-specific primers and identified the species only after sequence analysis [25,39]. In contrast, our current study utilized primers specifically detecting *T. orientalis*. The detection rate obtained in this study was lower than the two previous reports, which could be due to the number of samples tested and the area covered, including the Province of Laguna. A previous study in Cebu was unable to detect *Theileria* in cattle, and the authors suggested that it might be due to the absence of the tick vectors in the area (i.e., *Haemaphysalis* and *Hyalomma* spp.) [40]. Only the cattle tick *Rhipicephalus microplus* was observed in cattle in the study area [27,28]. This is similar to the study of Jirapattharasate et al. [43], in which they were only able to collect *R. microplus* ticks in their study in Thailand, where they detected *T. orientalis*. In a study in Vietnam, *T. orientalis* was detected in 10.6% of *R. microplus* ticks collected from cattle using PCR, suggesting the possible role of this tick in the transmission of *T. orientalis* [50]. Another study investigated the potential role of hematophagous flies and lice in the endemicity of *T. orientalis* in cattle farms in Australia, where ticks were not observed [51]. DNA of *T. orientalis* was detected in biting flies, midges, and lice from cattle, and the authors suggested that these blood-sucking arthropods might be able to serve as mechanical vectors of *T. orientalis*. These previous studies, together with our current findings, warrant further studies to determine the role of *R. microplus* as well as other external parasites of cattle present in the country in transmitting *T. orientalis*. Transplacental transmission of *T. orientalis* may also be implicated in the spread of the pathogen, but this mode of transmission reportedly occurs only at a low rate [52].

A lower detection rate for single infections was observed for *B. bigemina* (2.9%) and *B. bovis* (5.6%) than *T. orientalis* (14.1%). Lower field detections of *Babesia* compared to *Theileria* have been attributed to a lower number of circulating piroplasms in animals infected with the former [12]. *Theileria orientalis* was also more prevalent than the two *Babesia* spp. in previous studies in cattle in Thailand and Vietnam [43,44]. Weerasooriya et al. [44] suggested that the vectors of *T. orientalis,* which are three-host ticks, are more capable of infecting a higher number of cattle than vectors of *B. bigemina* and *B. bovis,* which are one-host ticks.

Concurrent infections of *Babesia* spp. and *T. orientalis* were detected with dual infections at a rate of 3.4% and 0.5% for infections with three species. This finding may suggest a common vector capable of transmitting both species of parasites [8] and thus warrants further investigation. Concurrent infections may also aggravate manifestations of the disease [53]. Animals with multiple infections may have a higher degree of anemia than infections with *T. orientalis* alone, as observed in a study of endemic piroplasms in Japan [54]. Furthermore, benign theileriosis may have a fatal outcome with co-infections because of the absence of cross-protection between the parasites [43].

Some of the cattle samples that tested positive in nested PCR for piroplasms (*Babesia/Theileria*) were negative in all species-specific PCR assays. This suggests the possibility of the presence of other species of piroplasm in the region. Belotindos et al. [25] suggested that the Philippine isolates of *Theileria* in their study could be a new species. Other studies in the Philippines were conducted to detect *T. annulata* and *B. ovata* in cattle in Cebu [40,55], and in water buffaloes in Bohol, but none of the samples tested positive [46]. Their presence in the country can only be ruled out, however, if other provinces are tested for these parasites [55]. 

The majority of the samples that were positive for any piroplasm were dairy cattle belonging to temperate breeds or crossed with tropical breeds. The lactation period in dairy animals can cause non-specific immunosuppression [49], which may be attributed to this finding. Bawm et al. [42] also found that crossbred cattle have a higher chance of acquiring *Babesia* than Zebu cattle. The Philippine native cattle and Zebu breeds, which are mainly utilized for beef and are known to be tick-resistant, were also included in this study. Similar to our previous studies on other tick-borne pathogens [27,28], piroplasms were also detected in the blood of these animals, albeit at a lower rate than that in dairy animals. This indicates the role of these tropical cattle breeds as potential reservoirs that can contribute to the maintenance of piroplasms under local conditions [28]. 

Clinical signs were not observed in the positive animals except for a few cattle with emaciation and some degree of lameness [27,28]. The absence of disease manifestations in most of the animals that tested positive suggests carrier status and endemic stability of the parasites. The animals in the area might have been exposed to the pathogens early in life, such that they already show little to no clinical signs [13]. However, these asymptomatic animals may still serve as a source of infection for ticks, becoming a major concern for the control of piroplasmosis [9,56]. When exposed to these tick vectors, naive or previously unexposed animals introduced in these areas may develop severe signs and life-threatening disease. Thus, infections with these blood parasites may lead to higher losses due to mortality and loss of productivity of the affected animals. Therefore, the identification of these chronic carriers is important in preventing the transmission of these pathogens to imported animals, which is part of development programs aimed at increasing farm production. 

As already mentioned above, only *R. microplus,* which was identified based on morphology [27], was observed from some of the animals that tested positive for piroplasms. Aside from the availability of hosts, variations in the prevalence of TBDs may also be attributed to the difference in the abundance of ticks in a particular location [57]. Therefore, the distribution of tick vectors and other potential vectors across municipalities should be considered in future studies. These vectors should also be subjected to PCR to detect piroplasms to verify their role in disease transmission.

## 5. Conclusions

In this study, two species of *Babesia* (*B. bigemina* and *B. bovis*) and *T. orientalis* were detected in large ruminants in four provinces in Southern Luzon, Philippines, through PCR. *Babesia bovis* and *T. orientalis* were detected in cattle from all four provinces, whereas *B. bigemina* was not detected in one province. Only *B. bigemina* and *B. bovis* were detected in water buffaloes in the Province of Quezon only. Concurrent infections with these piroplasms were also observed. This information warrants intensified control and prevention strategies for TBDs to support the livestock development programs in the country. 

Since the majority of the positive animals were asymptomatic, suggesting carrier status, the examination of the hematologic profiles of these animals may aid in assessing the impact of these pathogens on the health and possibly productivity of the affected animals. Future epidemiological studies should include a larger sample size and a wider study area to estimate the prevalence of these piroplasms, not only in the region, but in the entire country. The local transmission of *T. orientalis,* including the vector involved, should also be investigated. The influence of seasonal variation and tick distribution on the occurrence of these pathogens should also be examined.

## Figures and Tables

**Table 1:** Number and percent (%) of blood samples from cattle and water buffalo from selected provinces in Luzon, Philippines, that tested positive for piroplasms (*Babesia/Theileria*), based on nested PCR. *n* = number of tested samples.

	Cattle		Water Buffalo	
Province	*n*	No. (%) of Piroplasm-Positive	*n*	No. (%) of Piroplasm-Positive
Laguna	111	34 (30.6)	11	0
Batangas	120	55 (45.8)	8	0
Rizal	87	22 (25.3)	0	--
Quezon	94	12 (12.8)	89	3 (3.4)
Total	412	123 (29.9)	108	3 (2.8)

**Table 2:** Number and percent (%) of blood samples in cattle from selected provinces in Luzon, Philippines, that tested positive for *Babesia bigemina, Babesia bovis,* and/or *Theileria orientalis* based on species-specific PCR. *n* = total number of blood samples.

Pathogens Detected	No. (%) of Positive Cattle				
	Laguna *n* = 111	Batangas *n* = 120	Rizal *n* = 87	Quezon *n* = 94	Total *n* = 412
**One pathogen**					
*Babesia bigemina*	4 (3.6)	7 (5.8)	0	1 (1.1)	12 (2.9)
*Babesia bovis*	8 (7.2)	10 (8.3)	0	5 (5.3)	23 (5.6)
*Theileria orientalis*	12 (10.8)	22 (18.3)	20 (23.0)	4 (4.3)	58 (14.1)
*Subtotal*	24 (21.6)	39 (32.5)	20 (23.0)	10 (10.6)	93 (22.6)
**Two pathogens**					
*B. bigemina* + *B. bovis*	0	2 (1.7)	0	0	2 (0.5)
*B. bigemina* + *T. orientalis*	1 (0.9)	5 (4.2)	0	0	6 (1.5)
*B. bovis* + *T. orientalis*	0	4 (3.3)	2 (2.3)	0	6 (1.5)
*Subtotal*	1 (0.9)	11 (9.2)	2 (2.3)	0	14 (3.4)
**Three pathogens**					
*B. bigemina* + *B. bovis* + *T. orientalis*	1 (0.9)	1 (0.8)	0	0	2 (0.5)
*Subtotal*	1 (0.9)	1 (0.8)	0	0	2 (0.5)
Total	26 (23.4)	51 (42.5)	22 (25.3)	10 (10.6)	109 (26.5)

**Table 3:** Host attributes of cattle and water buffaloes from selected provinces in Luzon, Philippines, that tested positive for piroplasms based on species-specific PCR. *n* = total number of samples.

Host Attribute	*n*	Number (%) Positive for at Least One Species of Piroplasm	*p*-Value
**Species**			
Cattle	412	109 (26.5)	<0.0001 *
Water buffaloes	108	3 (2.8)	
**Type**			
Dairy	275	87 (31.6)	<0.0001 *
Meat	177	24 (13.6)	
Draft	68	1 (1.5)	
**Sex**			
Male	80	11 (13.8)	0.0654
Female	440	101 (23.0)	

* Statistically significant at *p <* 0.05.

## Data Availability

The nucleotide sequences of selected isolates were deposited in the DNA Data Bank of Japan (accession numbers LC684833 to LC684846).

## Supplementary Materials

The following are available online at https://www.mdpi.com/article/10.3390/microorganisms10040678/s1; Table S1. Primers used in the study for the amplification of piroplasms (*Babesia/Theileria* spp.), specifically, *Babesia bigemina, B. bovis,* and *Theileria orientalis;* Table S2. PCR conditions used in the study for the amplification of piroplasms (*Babesia/Theileria* spp.), specifically, *Babesia bigemina, B. bovis,* and *Theileria orientalis.*
