# Supplementary material for: Molecular Detection and Characterization of Babesia and Theileria in Cattle and Water Buffaloes from Southern Luzon, Philippines
Source: Microorganisms. 2022 Mar 22;10(4):678. doi: 10.3390/microorganisms10040678 (PMC9025510; doi:10.3390/microorganisms10040678)
Supplement: Supplementary file 1 [file microorganisms-10-00678-s001.zip › microorganisms-1637629-supplementary.pdf]

Supplementary Materials:

Table S1. Primers used in the study for the amplification of piroplasms (*Babesia/Theileria* spp.), specifically, *Babesia bigemina, B. bovis* and *Theileria orientalis.*

Organism and Type of PCR	Target Gene	Primer Name	Sequence (5′ → 3′)	Expected Size (bp)	References
Piroplasms (*Babesia/ Theileria* spp.) Nested	*18s rRNA*	piro18s forward 1	GGTGAAACTGCGAATGGCTC		[29]
		piro18s reverse 1	AAGTGATAAGGTTCACAAAACTT	1600	
		piro18s forward 2	TGGCTCATTACAACAGTTATA		
		piro18s reverse 2	CGGTCCGAATAATTCACC	1500	
Babesia bigemina Conventional	*apical membrane antigen-1*	AMA-1 forward	TACTGTGACGAGGACGGATC		
		AMA-1 reverse	CCTCAAAAGCAGATTCGAGT	211	[30]
Babesia bovis Nested	*spherical body protein- 4*	SBP-4 forward	GAAATCCCTGTTCCAGAG		[31]
		SBP-4 reverse	TCGTTGATAACACTGCAA	907	
		SBP-4 forward	GAAATCCCTGTTCCAGAG		
		SBP-4 reverse	TCGTTGATAACACTGCAA	503	
Theileria orientalis Conventional	*major piroplasm surface protein*	MPSP forward	CTTTGCCTAGGATACTTCCT		
		MPSP reverse	ACGGCAAGTGGTGAGAACT	776	[32]

Table S2. PCR conditions used in the study for the amplification of piroplasms (*Babesia/Theileria* spp.), specifically, *Babesia bigemina, B. bovis,* and *Theileria orientalis.*

Target Gene	PCR Condition
18s rRNA	1st round: $\frac{95^{\circ}C}{9\,min} \rightarrow \left[\frac{94^{\circ}C}{30\,s} - \frac{55^{\circ}C}{30\,s} - \frac{72^{\circ}C}{90\,s}\right] 35x \rightarrow \frac{72^{\circ}C}{7\,min} \rightarrow 10^{\circ}C \;\; \infty$
	2nd round: $\frac{95^{\circ}C}{9\,min} \rightarrow \left[\frac{94^{\circ}C}{30\,s} - \frac{53^{\circ}C}{30\,s} - \frac{72^{\circ}C}{90\,s}\right] 35x \rightarrow \frac{72^{\circ}C}{5\,min} \rightarrow 10^{\circ}C \;\; \infty$
apical membrane antigen-1	$\frac{95^{\circ}C}{5\,min} \rightarrow \left[\frac{95^{\circ}C}{30\,s} - \frac{68^{\circ}C}{1\,min} - \frac{72^{\circ}C}{1\,min}\right] 35x \rightarrow \frac{72^{\circ}C}{10\,min} \rightarrow 10^{\circ}C \;\; \infty$
spherical body protein- 4	1st round: $\frac{95^{\circ}C}{5\,min} \rightarrow \left[\frac{94^{\circ}C}{1\,min} - \frac{55^{\circ}C}{1\,min} - \frac{72^{\circ}C}{1\,min}\right] 35x \rightarrow \frac{72^{\circ}C}{10\,min} \rightarrow 10^{\circ}C \;\; \infty$
	2nd round: $\frac{95^{\circ}C}{5\,min} \rightarrow \left[\frac{94^{\circ}C}{1\,min} - \frac{55^{\circ}C}{1\,min} - \frac{72^{\circ}C}{1\,min}\right] 35x \rightarrow \frac{72^{\circ}C}{10\,min} \rightarrow 10^{\circ}C \;\; \infty$
major piroplasm surface protein	$\frac{94^{\circ}C}{10\,min} \rightarrow \left[\frac{94^{\circ}C}{1\,min} - \frac{58^{\circ}C}{1\,min} - \frac{72^{\circ}C}{1\,min}\right] 35x \rightarrow \frac{72^{\circ}C}{4\,min} \rightarrow 10^{\circ}C \;\; \infty$
